# Homocysteine Solution-Induced Response in Aerosol Jet Printed OECTs by Means of Gold and Platinum Gate Electrodes

**DOI:** 10.3390/ijms222111507

**Published:** 2021-10-25

**Authors:** Pasquale D’Angelo, Mario Barra, Patrizia Lombari, Annapaola Coppola, Davide Vurro, Giuseppe Tarabella, Simone Luigi Marasso, Margherita Borriello, Federico Chianese, Alessandra F. Perna, Antonio Cassinese, Diego Ingrosso

**Affiliations:** 1 IMEM-CNR, Parco Area delle Scienze 37/A, I 43124 Parma, Italy; pasquale.dangelo@imem.cnr.it (P.D.); davide.vurro@imem.cnr.it (D.V.); simone.marasso@imem.cnr.it (S.L.M.); 2 CNR-SPIN, c/o Dipartimento di Fisica “Ettore Pancini”, P.le Tecchio 80, 80125 Naples, Italy; mario.barra@spin.cnr.it; 3 Department of Precision Medicine, University of Campania “Luigi Vanvitelli”, via L. De Crecchio 7, 80138 Naples, Italy; patrizia.lombari@unicampania.it (P.L.); annapaola.coppola@unicampania.it (A.C.); margherita.borriello@unicampania.it (M.B.); diego.ingrosso@unicampania.it (D.I.); 4 Department of Translational Medical Science, University of Campania “Luigi Vanvitelli”, via Via Pansini, Bldg., 80131 Naples, Italy; 5 Camlin Italy Srl, Via Budellungo 2, 43124 Parma, Italy; giuseppe.tarabella@camlingroup.com; 6 Physics Department, University of Naples “Federico II”, P.le Tecchio, 80, 80125 Naples, Italy; federico.chianese@unina.it; 7 Istututo Nazionale di Fisica Nucleare, Sezione di Napoli, P.le Tecchio, 80, 80125 Naples, Italy

**Keywords:** homocysteine, organic electrochemical transistors, point-of-care testing, cardiovascular risk

## Abstract

Homocysteine (Hcy) is a non-protein, sulfur-containing amino acid, which is recognized as a possible risk factor for coronary artery and other pathologies when its levels in the blood exceed the normal range of between 5 and 12 μmol/L (hyperhomocysteinemia). At present, standard procedures in laboratory medicine, such as high-performance liquid chromatography (HPLC), are commonly employed for the quantitation of total Hcy (tHcy), i.e., the sum of the protein-bound (oxidized) and free (homocystine plus reduced Hcy) forms, in biological fluids (particularly, serum or plasma). Here, the response of Aerosol Jet-printed organic electrochemical transistors (OECTs), in the presence of either reduced (free) and oxidized Hcy-based solutions, was analyzed. Two different experimental protocols were followed to this end: the former consisting of gold (Au) electrodes’ biothiol-induced thiolation, while the latter simply used bare platinum (Pt) electrodes. Electrochemical impedance spectroscopy (EIS) analysis was performed both to validate the gold thiolation protocol and to gain insights into the reduced Hcy sensing mechanism by the Au-gated OECTs, which provided a final limit of detection (LoD) of 80 nM. For the OECT response based on Platinum gate electrodes, on the other hand, a LoD of 180 nM was found in the presence of albumin-bound Hcy, with this being the most abundant oxidized Hcy-form (i.e., the protein-bound form) in physiological fluids. Despite the lack of any biochemical functionalization supporting the response selectivity, the findings discussed in this work highlight the potential role of OECT in the development of low-cost point-of-care (POC) electronic platforms that are suitable for the evaluation, in humans, of Hcy levels within the physiological range and in cases of hyperhomocysteinemia.

## 1. Introduction

In recent years, the rising interest in Bioelectronics has mainly been supported by the astonishing development of new electronic devices based on conducting organics (i.e., conjugated polymers). In several cases, indeed, these compounds are able to exhibit a mixed ionic-electronic conduction mode, which makes them particularly effective for their proper interfacing/interaction with biosystems [1]. 

A number of organic electronic devices in a transistor-like configuration, such as EGOFETs and OECTs, have been shown to be effective for the detection of biomolecules in biosystems through user-friendly and cost-effective approaches [2,3]. In particular, OECTs have emerged as versatile tools that are able to implement a Lab on Chip approach (allowing, for instance, the monitoring of biomolecules’ properties [4] and the properties of cells’ physiology [5], or even of neuromorphic function [6]). Such devices are able to convert changes in bioanalyte concentrations into measurable electronic current variations, preserving sufficient accuracy, reproducibility and sensitivity. In particular, the OECT standard architecture consists of a PEDOT:PSS (poly(3,4-ethylenedioxythiophene)-polystyrene sulfonate) channel, filling the gap between two metal electrodes (source and drain), which is interfaced to an electrolyte containing the third electrode, named the gate. OECTs can work in a liquid medium for prolonged time at operating voltages well below 1 V. These devices implement an ion-to-electron transduction with a marked amplifying capability, since, during their operation, the ionic species in the electrolyte are reversibly forced, by the gate voltage, towards the PEDOT:PSS channel, producing its charge de-doping and the related decrease in conductivity. The surfaces of the main OECT components, especially the gate, can be bio-functionalized to improve the final selectivity towards the desired analyte (e.g., glucose, dopamine, or DNA) via specific electrochemical or biological interactions [7]. The combination of all these features has contributed to the rising interest in this sensing platform for use in potential diagnostic tools in laboratory medicine or as POC testing devices. Although the adoption of functionalization protocols is actually a crucial step for the operation in real bio-organic fluids, such as serum/plasma, urine and saliva, it is, however, possible to design strategies targeted to tailoring a proper sensing operation where, for example, false negative results are minimized upon the choice of suitable electrodes (to be eventually functionalized) being inert towards different interfering species [8]. More generally, the experimental design may benefit from the knowledge of the main features of both the biologic matrix [9] and the bioanalyte [10] to be detected by the OECT biosensor. In this respect, the features of some molecules that are of great interest in medicine may help to establish simple and cost-effective methods aimed at studying their physiochemical characteristics, while offering a tool for their determination in physiological environments. 

**Figure 1:**
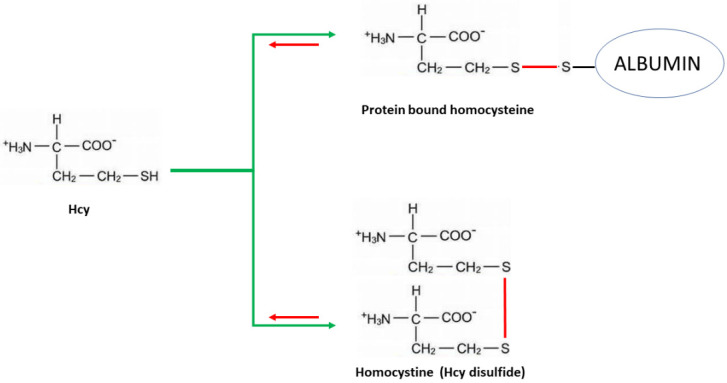
Various forms of Homocysteine present in vivo. The albumin adduct oxidized derivative (protein bound) is, by far, the most prevalent, while the disulfide is normally almost negligible, except under some pathological conditions (e.g., homocystinuria). Although some authors hypothesized a role for either the protein-bound oxidized or the free forms, in clinical practice, total homocysteine is detected (tHcy), i.e., the sum of all forms, since current laboratory methods have been set up by accomplishing a preliminary reducing step (red arrows) prior to analytical procedures for quantitation.

Homocysteine (Hcy), a non-protein, sulfur-containing amino acid (see chemical structure reported in Figure 1) is the demethylated methionine derivative, occurring in the methionine-homocysteine cycle. Although, as an amino acid, Hcy is endowed with the presence of both one amino and one carboxyl group, it is not utilized in protein biosynthesis because it is not encoded by any codon-anticodon system. It is important to mention that, due to its high pKa, the free sulfidryl group on the Hcy side chain is easily oxidized at a physiological pH [11]. Hence, because of the strong reactivity of this thiol group, only about 1 ÷ 2% of Homocysteine is usually present in its free reduced form. Conversely, most of Hcy is carried in circulation in the form of a heterodimer that is mostly bound to the cysteine of serum albumin, while only a minimum is in the homodimer protein-free form (homocystine). Homocystine-mixed disulfides with free homocysteine or cysteine represent less than 15% of the total Hcy in serum. The Hcy-protein covalent disulfide adduct is by far the most abundant (for instance, at least 80% of homocysteine is bound to albumin) (Figure 1). 

Normal levels of tHcy in serum range between 5 and 10–12 μmol/L (for female and male subjects, respectively), while hyperhomocysteinemia (HHcy) is an excess of Hcy, normally classified as mild (up to 15 μmol/L), moderate (16–30 μmol/L), intermediate (31–100 μmol/L), and severe (>100 μmol/L) [12,13,14]. HHcy is generally recognized as an independent cardiovascular risk factor for increased arterial and venous thrombophilic states [15], as well as a number of other pathologies [16]. The importance of Hcy is related to cardiovascular risk in general, but especially in hypertensive individuals, in whom folate supplementation may be an important measure to be explored [17,18]. 

Hcy quantitation in serum or plasma matrices is a part of the thrombophilic risk profile evaluation, and is generally achieved using high-performance liquid chromatography (HPLC) or immunometric routine methods [19]. More recently, optical and electrochemical methods were proposed, although they have not yet been introduced in routine practice [20].

**Figure 2:**
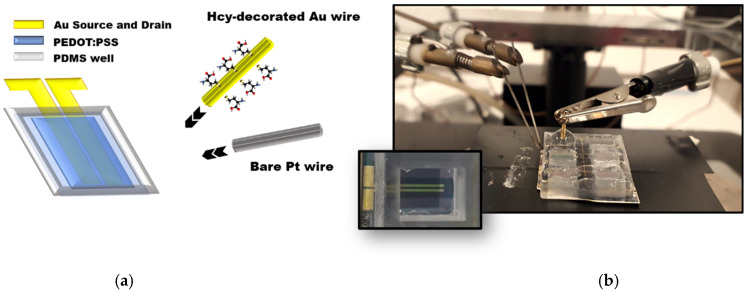
(**a**) Sketch of the OECT layout with Hcy-decorated Au- and bare Pt-gated electrodes used in this study. (**b**) Photograph of the OECT device connected to the probe-tip terminals (source and drain), showing the liquid electrolyte confined in the PDMS well and the Hcy-decorated gold gate electrode immersed in it; the inset shows a top-view picture of the real OECT.

In this work, following an innovative approach, we show how tHcy (both protein-bounded and free forms) can be, in principle, detected by an OECT (Figure 2a,b) featuring gold and platinum gate electrodes [21]. OECT devices consist of active channels, deposited by a 3D printing technique, namely the Aerosol Jet printing (AJP), which is the most promising Additive Manufacturing technique for 3D-printed electronics. Indeed, compared with conventional InkJet printing, AJP shows an improved resolution with a larger variety of printable materials. Therefore, the AJP technique is particularly suitable for mass production manufacturing [22].

In particular, reduced Hcy (rHcy) could be detected by an OECT-gated gold electrode, owing to the interaction between the bio-thiol Hcy and the bare Au surface (Figure 2b). On the other hand, oxidized Hcy, prevalently formed in the presence of the most abundant serum albumin, may be distinguished from Hcy free forms by using a bare platinum electrode and exploiting the electrochemical oxidation of amines at the Pt surface [23]. It is worth remembering that oxidized protein-bound Hcy may almost adequately mimic a physiological microenvironment [24]. Both the Au- and Pt-gated OECTs investigated here show a dynamic sensing range comprising the normal free Hcy concentration levels in serum. 

## 2. Results

### 2.1. Electrochemical Impedance Spectroscopy (EIS) Experiments on Thiolated Gold SPE

**Figure 3:**
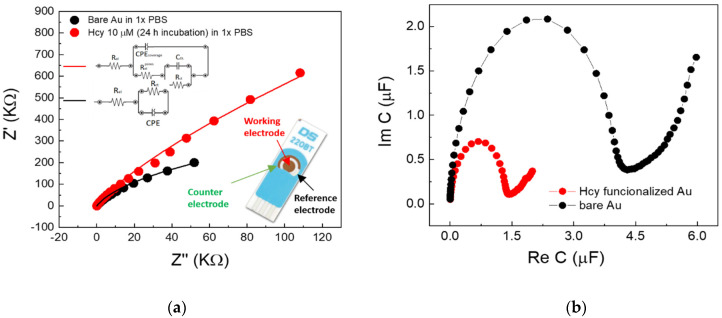
(**a**) Nyquist plot for the gold working electrodes of a SPE before (black symbols) and after (red symbols) their incubation in Hcy:HCl:PBS solutions (HCy concentration of 10 µM); continuous lines are the related fitting curves for the proposed equivalent circuit analysis; the inset shows a real picture of the commercial SPE used in this experiment. (**b**) Complex capacitance plot of the bare (black symbols) and covered (red symbols) electrodes calculated from the acquired complex impedances.

A comparative EIS analysis, carried out by acquiring the Nyquist plot, i.e., the imaginary part of the complex impedance (Z(ω) = Z′ + iZ″) as a function of its real part, for a bare (black symbols in Figure 3a) and an incubated gold electrode (red symbols in Figure 3a), was conducted with the aim of showing that the gold incubation in rHcy solutions promotes the thiolation of the electrode. In particular, EIS measurements were performed in a physiological electrolyte (1× PBS, pH 7.3) upon incubation of the Au working electrode of a screen-printed electrode (SPE) by a Hcy/HCl/PBS solution. For this experiment, the concentration of Hcy was fixed at 10 µM, i.e., in the middle of the investigated Hcy concentration range. 

The acquired curves show specific differences both in their shape and magnitude, suggesting that the incubation process and the related electrode coverage were efficiently carried out. This occurrence was further investigated by an equivalent circuit analysis, where proper models (see the equivalent circuits reported in the inset of Figure 3a) were used to characterize the electrochemical properties of electrolyte/interface pairs, as well as the features of the surface coverages.

The whole set of the extracted fitting parameters is reported in Table 1. While, as expected, R_el_ (related to the electrolyte conductivity) is comparable in both cases and shows a magnitude of few tens of Ohm, the analysis of the other parameters provides evidence of the expected thiolation (see the Discussion Section 3).

The assessed ideality factor (n) of 0.944 for the uncovered gold electrode indicates the formation of a double layer at the gold/PBS interface. On the other hand, the elevated R_ct_ value (in the order of 1 MΩ) is fully compatible with the fact that, at the interface between a polarizable electrode and a saline medium, charge transfer phenomena are minimal, being due only to residual impurities between grain boundaries. In addition, the complex capacitance plot, calculated from the acquired complex impedance Z as C = 1/jωZ, is reported in Figure 3b.

### 2.2. Au-Gated OECTs

Providing that EIS measurements corroborate the efficiency of the thiolation protocol (as discussed in Section 3), we tested the gold electrode decoration upon SH-Au interaction as a probe for the detection of free rHcy by OECTs. To this end, gold wires were immersed for 24 h in Hcy:HCl:PBS solutions at different concentrations of both Hcy and HCl, leaving a ratio of 1:1 between Hcy and HCl (see methods Section 4.5). 

**Figure 4:**
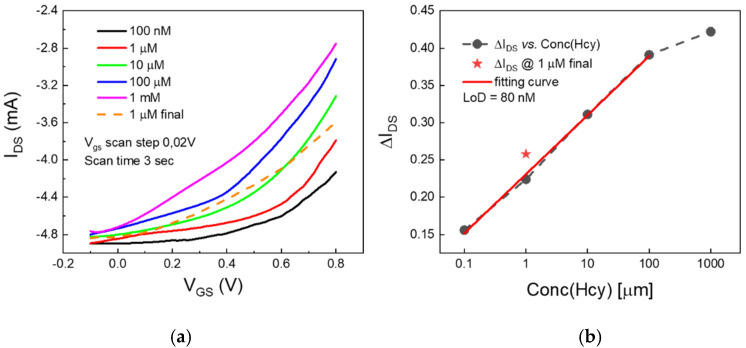
(**a**) (solid lines) OECT transfer curves measured after the gate gold electrode incubation in Hcy:HCl:PBS solutions with progressively-increased Hcy concentrations (from 100 nM (black curve) to 1 mM (magenta curve); the dotted orange curve was measured at the end of this set of measurements and using a gold electrode incubated at 1µM of Hcy. (**b**) The IDS modulation parameter, defined as [(IDS(@VGS = 0.8 V) − (IDS(@VGS = 0.1 V)]/(IDS(@VGS = 0.1 V), extracted from all the recorded transfer curves in panel (**a**), as a function of the Hcy concentration in Hcy:HCl:PBS solutions.

Transfer curves, reported in Figure 4a, were recorded using PBS as an electrolyte for OECTs gated by gold electrodes that were previously incubated at different rHcy concentrations. The investigated Hcy range, from 100 nM to 1 mM, was selected in such a way as to comprise concentrations below the physiological range and above that of severe HHcy. According to our analysis, we found that the OECT response is more efficient (i.se. larger I_DS_ modulation values) as the rHcy concentration in the incubation solution increases. An enhancement of the gate current values on Au electrode incubation was also observed (Figure S3).

As reported in Figure 4b, the OECT sensing response can be described by a linear-log plot of the modulation parameter ΔI_ds_ as a function of rHcy concentration in Hcy:HCl:PBS solutions (calibration curve). The analyzed Hcy concentration range fell within the sensor dynamic range, except for the highest Hcy concentration of 1 mM. Therefore, from a linear regression of the calibration curve, it was possible to define the sensor LoD (i.e., the lowest measurable analyte concentration that could be detected in sample) with a high confidence level. In detail, following the standard rule by IUPAC, the LoD is defined as follows:

LoD = 3σ/S(1)

where σ is the standard deviation of the response (i.e., the standard deviation of the y-intercept of the regression line) and S is the slope of the regression line (see Materials and Methods Section 4.5 for more details). In our case, the calculated LoD value of 80 nM is comparable to that of free-Hcy base level (1–2% of 5 ÷ 12 µM). 

### 2.3. Pt-Gated OECTs

**Figure 5:**
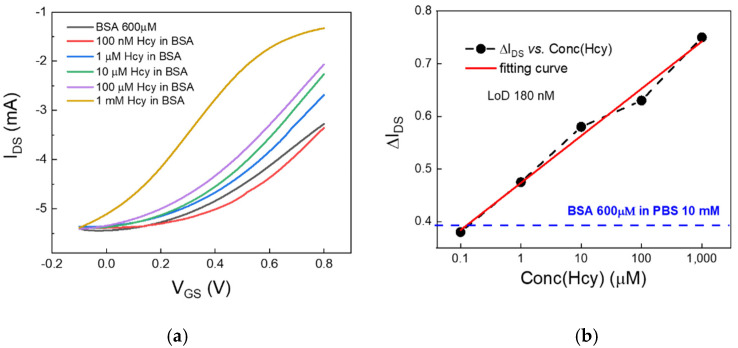
(**a**) OECT transfer curves measured with a platinum electrode in albumin-based solutions (at a fixed BSA concentration of 600 µM) at different Hcy concentrations (from 100 nM to 1 mM). (**b**) Lin-log plot of the modulation parameter ΔI_DS_, extracted from all the recorded transfer curves in panel (**a**), as a function of the Hcy concentration in BSA:PBS solutions. The dashed blue line represents the modulation parameter for the blank measurement (BSA in PBS).

Hcy-based solutions, consisting of Hcy dissolved at various concentrations (from 100 nM to 1 mM) in a physiologic-like microenvironment made of a PBS:BSA (10 mM:600 µM), were used as gate electrolytes for Pt-gated OECTs. Since the neutral electrolytic environment favors the reactivity of the thiol group, oxidative reactions between albumin and Hcy were expected to promote Hcy–BSA binding to a large extent and in a rapid manner [25]. Therefore, the Pt-based OECTs operated in a simil-physiological microenvironment made of some free Hcy and an excess of albumin (BSA–Hcy). The resulting transfer curves are reported in Figure 5a. Interestingly, increasing levels of BSA-Hcy in the electrolytic solution were found to promote an enhanced I_DS_ current modulation. 

The corresponding calibration curve, consisting of the modulation parameter as a function of the Hcy concentration, is reported in Figure 5b. The nominal LoD by Pt-gated OECT, calculated from the calibration curve using Equation (1), was 180 nM. This value again represents the upper limit for the actual free Hcy concentrations, since a non-negligible fraction of Hcy is at least expected to be involved in the formation of albumin-aggregated forms. It is worth noting that the low modulation parameter assessed in the case of bare BSA-based electrolyte indicated that Hcy albumin-aggregate forms and eventual free Hcy fractions, not involved in the formation of Hcy-albumin aggregates, may be unambiguously detected by OECTs.

Finally, for the sake of completeness, platinum-gated OECT were also investigated while using directly rHcy:HCl:PBS solutions (i.e., those used for gold thiolation) as electrolyte (Figure S2a). In this case, data analysis (Figure S2b) indicates that this acidic electrolyte environment produces a remarkable increase in the LoD by one order of magnitude (LoD = 2.5 µM) in comparison with the Pt-gated OECT investigated in Hcy:BSA:PBS electrolytic solutions. A reduced dynamic range, represented by the linear range in the lin-log plot of the sensor response, was also observed (Figure S2b).

## 3. Discussion

In this work, multiple electrical tests (listed in the following Table 2 for the sake of completeness), based on screen-printed electrodes or OECT with different gating conditions, were performed in order to define a suitable experimental strategy to investigate the homocysteine content in different types of solutions. 

The use of gold or platinum electrodes, in particular, in OECTs allowed the effective detection of Hcy both in the reduced free form (rHcy) and in the presence of albumin-bound Hcy, with this being the most abundant oxidized Hcy-form mimicking a physiological microenvironment.

In the preliminary EIS experiments on thiolated Gold SPE (Section 2.1), the choice of the PBS:HCl solution as the liquid medium for the gold thiolation by free rHcy was justified by the fact that low pH levels generally stabilize against oxidation of this molecule [26]. Given the zwitterionic character of the molecule, the protonation of the amino group at low pH values generates repulsive interactions that limit the oxidative processes of free Hcy molecules. Hence, during the incubation process, the free Hcy concentration in the acidic solution is reasonably maintained around its nominal level, or at least its reduction process is less effective with respect to the case of Hcy in neutral solutions. Nevertheless, it should be noted that the formation of the disulfide occurs through the formation of a thiolate ion [27]. On the other hand, the thiolate ion is probably destabilized by low pH. According our present results, the gold/PBS interface can be modeled using the Randles equivalent circuit (see the inset of Figure 2a), where an electrical resistor, R_el_, representing the bulk electrolyte, is in series with a parallel circuit between the Constant Phase Element (CPE) and the charge transfer resistor, R_ct_. The last parameter takes into account the role of charge transfer mechanisms due to impurities on the (polycrystalline) gold surface [28]. CPEs are commonly used to describe the behavior of real systems and the related impedance can be expressed as Z_CPE_ = 1/(jω)^n^Y_0_, where n, the ideality factor, ranges between 0 and 1 (for *n* = 0, it corresponds to the impedance of a resistor, while for *n* = 1, the CPE corresponds to an ideal capacitor). 

As clearly shown in Figure 3a (see the black line), the Randles equivalent circuit adequately fits the behavior of the uncovered (bare) gold surface. Conversely, it completely fails to model the curve achieved for the incubated electrode (Figure S1). In this case, indeed, the gold-Hcy/PBS interface requires an alternative model that is also able to account for the electrode coating. Starting from the circuit model, which is valid for an ideal coverage condition, where R_el_ is simply in series with a capacitor, real case modifications firstly require the replacement of the capacitor with a CPE, taking into account the surface roughness. Then, it is needed an implementation of the above series in a more complex form, where an element describing the contribution of the uncovered surface (generally pores of different size and surface distribution) is added in parallel to the CPE. This element consists of a series linking the electrolyte resistance through the pores (R_el_^pores^, ideally infinite if the coverage is free of pores), as well as a parallel given by the double layer capacitance of the uncovered metal surface (C_DL_) and a Faradaic impedance (which, in the simplest form, can be a charge transfer resistance). The red line in Figure 3a demonstrates that this model is able to describe the behavior of the incubated surface in a much more convincing way.

Table 1 reports the parameters extracted for the incubated gold surface and indicates that: (i) in this case also, the ideality factor (*n* = 0.978) value agrees with the formation of a double layer, as expected for non-faradaic systems (which gold/PBS interfaces actually are), which is ascribable to the covered portion of the electrode surface; (ii) the high R_el_^pores^ value is compatible with the presence of uncovered regions on the electrode surface; (iii) the R_ct_ value is even higher (3.5 times) than that found for the uncovered electrode, suggesting that the coating of a large portion of the surface electrode makes it possible to reduce the impurity-related faradaic contribution. 

Figure 3b highlights the difference in total capacitance for Hcy-covered and bare gold electrodes and allows the estimation of the total capacitance at the bare gold electrode (calculated as the intercept value between the real axis and the semicircle [29]), which is about 4 µF. Although such representation is controversial and tricky in terms of estimating the double layer capacitance in cases of defective real coatings described by modified Randles equivalent circuits, the Nyquist plot, reported in Figure 3a for the Hcy-covered Au electrode, qualitatively indicates that its total capacitance is enhanced (i.e., it is expected to be higher than 4 µF). 

The experiments with Au-gated OECTs (Section 2.2) show a better response with increasing rHcy concentrations (Figure 4a). This finding may be ascribed to the progressive increase in the electrode capacitance in PBS upon the incubation of the electrode in increasingly highly concentrated Hcy solutions [30]. In addition, the observed enhancement of the gate current values upon incubation of the Au electrode (Figure S3) coherently suggests that the potential drop at the gate electrode/electrolyte interface is actually lowered. In these conditions, a better coupling between the electrolyte and the PEDOT:PSS channel (i.e., larger corresponding gate-induced potential drops) was also obtained.

By calculating the LoD value of our OECT apparatus for rHcy detection; a value of 80 nM was found, with this value being comparable to that of the free-Hcy base level (1–2% of 5 ÷ 15 µM). It is remembering that, even though the acidic conditions were expected to limit the reactivity of the thiol group, it is likely that a free Hcy fraction experienced an auto-oxidation process. This means that the nominal free Hcy concentration value in the considered incubation solutions should represent an upper limit for the real Hcy concentrations. This may happen mostly at the lowest concentrations, where the acidic content of the incubation solution is reduced as a consequence of increasing dilutions of the stock solution in physiological PBS. Finally, our results suggest that a better reproducibility of the sensor response caused by repetitions of the gate voltage sweeps can be achieved by possibly reducing the applied V_GS_ voltage window range. This should be ascribed to the fact that the strength of the SH-Au bond in acidic conditions is not expected to be particularly strong [31] and that the bond may be easily disrupted by repeated measurement.

Furthermore, in the experiment carried out with albumin solution, an increasing level of Hcy promoted an enhanced I_DS_ current modulation (Figure 5a). In this case, however, this finding is basically ascribable to the occurrence of Faradaic reactions at the Pt electrode, which may be mainly related to free Hcy molecules. In fact, it is, for instance, well known that the oxidation of aliphatic amines in platinum can determine the formation of cyanide species which, in turn, can be also adsorbed on the electrode surface, independently of the acidic and basic conditions of their liquid environment [32].

The larger variations of the modulation parameter, in comparison with those achieved for the Au-gated OECT, were due to the higher efficiency of the platinum electrode in sustaining the gate potential via faradaic currents generated upon red-ox reactions at the same electrode. As discussed above, conversely, gold is inert in saline solutions and tends to experience the formation of an EDL, which causes a remarkable gate voltage drop in the electrolyte, i.e., a weaker coupling between the gate electrode and the device channel [33].

The use of Hcy:HCl:PBS solutions as electrolytes (Figure S2a) showed an increase in the LoD (LoD = 2.5 µM) in comparison with the Pt-gated OECT investigated in Hcy:BSA:PBS electrolytic solutions (LoD 180 nM). Since HCl does not significantly affect the OECT modulation in the presence of saline PBS solutions (Figure S4), the observed LoD increase may be explained by invoking the expected protonation of the amine groups, which lowers the concentration of free Hcy molecules and consequently reduces the contribution of Faradaic reactions at the electrode. It should be also remembered that protonation generates repulsive forces between protonated Hcy molecules and the gate electrode (positively biased). As a whole, this is an indirect confirmation of the fact that, in neutral environments (e.g., in Hcy:BSA:PBS), the device response (i.e., I_DS_ modulation) is actually ruled by the oxidative process involving free Hcy at the platinum electrode. In conclusion, the estimated limit of detection (LoD) values is in the range between 80 and 180 nM, and thus, demonstrates the potential of OECT devices for the detection of Hcy in both the physiological range and under hyperhomocysteinemia conditions. Further studies involving specific functionalization protocols for metallic and/or carbon-based gate electrodes will aim to test the response of OECTs in the presence of biological samples in order to confirm their sensibility and to evaluate their selectivity towards a large set of interfering species that are expected to be contained in complex biological matrices. 

In conclusion, these results strengthen the potential application of OECT-based methods for the detection of Hcy in biomedicine. Although future work is still required, the approach introduced here could open the way for the future development of POC electronic platforms for Hcy detection, realized on different types of substrates, by relatively low-cost techniques and working at very low voltages (<1 V). The availability of this user-friendly tool could make possible the starting of high-throughput screening campaigns focused on the analysis of Hcy with reference to different pathological conditions. It was pointed out that the determination of tHcy, including all fractions included in Figure 1, is the general strategy for evaluating the levels of this amino acid in biological fluids, as established by the guidelines. Consistently, all current methods used in the clinical laboratory proceed through a reduction step, which basically, lacking selectivity toward individual fractions, allows the detection of all species, at once, as tHcy. However, a number of reports showed that each Hcy fraction has the potential to trigger biological events of specific pathophysiological meaning [34]. For example, protein-bound Hcy was found to exert specific immunological activities [35], while free Hcy was shown to be involved in other mechanisms. OECT, although providing for the determination of tHcy as the sum of individual forms, has the potential to discriminate between protein bound and non-protein bound forms, thus providing additional information of pathophysiological relevance [36,37,38].

## 4. Materials and Methods

### 4.1. Chemicals 

D,L Homocysteine (Hcy), Bovine serum albumin (BSA), Ethylene Glycol (EG), 3-glycidoxypropyltrimethoxysilano (GOPS), H_2_SO_4_ and Sylgard 184 polydimethyl siloxane (PDMS) were purchased from Sigma Aldrich (3050 Spruce St, Saint Louis, MO, USA) Water dispersion of PEDOT.PSS (Clevios PH1000) was purchased from Heraus (Heraeus Holding GmbH Heraeusstraße 12-14 D-63450, Hanau, Germany). Phosphate-buffered saline (PBS) tablets were purchased from Thermo Fisher (Thermo Fisher Scientific, 168 Third Avenue, Waltham, MA, USA). Solvents, isopropyl alcohol (IPA), acetone and hydrochloric acid (HCl), were purchased from CARLO ERBA Reagents S.r.l. (Via R. Merendi, 22—20007, Cornaredo (MI), Italy).

### 4.2. OECT Fabrication

Gold source and drain contacts were fabricated on a standard thermal SiO_2_/Silicon wafer using a typical photolithographic procedure described elsewhere [39]. The PEDOT:PSS active layer was deposited using a commercial Aerosol jet Printer (AJP 200, Optomec Inc., Albuquerque, NM, USA) equipped with an ultrasonic atomizer (UAMax).

PEDOT:PSS dispersion was first sonicated for 30 min, then filtered using a 0.45 μm PES syringe filter (LLG Labware) and mixed with both 5% of Ethylene glycol (acting as secondary dopant) and 1% GOPS (acting as grafting component). The as-prepared solution was diluted in Milli-Q water at a 1:1.5 volume ratio and placed into the ultrasonic vial. The printing process was carried out with a 200 µm nozzle at a speed of 5 mm/s. During this process, sheath gas and carrier gas flows were set 30 and 25 SCCM, respectively, while the plate temperature was set 60 °C [40]. The deposited PEDOT:PSS layer, defining a device channel having width W = 5 mm and channel length L = 200 µm, was thermally annealed at 120 °C for 30 min to improve the related conducting properties. Finally, a PDMS well was stacked over the channel through irradiation of the bottom side of the PDMS well with an UV radiation (NOVASCAN UV-O_3_ cleaner) for few seconds, in order to promote the reservoir adhesion on SiO_2_. The final device layout is reported in Figure 2b.

### 4.3. Hcy Solution Preparation

A stock solution was prepared by dissolving Hcy at 10 mM final concentration in 1 M HCl in PBS. For both gold wire incubation and electrical measurements by bare Pt electrodes, Hcy was diluted in PBS at different concentrations (from 100 nM to 1 mM) and used freshly just after preparation.

### 4.4. SPE Functionalization and Electrochemical Measurements

Commercial SPEs (model 220BT, purchased from Metrohm, (Metrohm Italiana, Origgio (VA), Italy), composed of gold working and counter electrodes and an Ag reference electrode, were cleaned with methanol and subsequent cyclic voltammetry (CV) cycles in H_2_SO_4_ 0.1 M. SPEs were incubated with a single drop (50 µL) of Hcy:HCl:PBS solution (Hcy concentration of 10 μM) for 24 h at 4 °C. Only the working electrode was coated by Hcy solution during the incubation process.

After incubation, electrodes were washed with PBS twice. EIS measurements (Nyquist and complex capacitance plots) were collected in 1× PBS by means of an electrochemical potentiostat/galvanostat (PalmSENS4- Randhoeve 221, 3995 GA, Houten, The Netherlands). To this end, an ac probe signal, with amplitude of 10 mV and a sweeping the frequency between 0.1 Hz and 100 KHz, was utilized.

### 4.5. Gold Wire Functionalization and Electrical Measurements

Gate electrodes were fabricated starting from a 99.99% pure gold wire (purchased from VWR), first cut in 5 mm length pieces, then washed in hot acetone and IPA and, finally, immersed in the as-prepared Hcy:HCl:PBS solutions for 24 h at 4 °C to achieve the binding of free Hcy molecules over the gold surface. Finally, the incubated electrodes (Figure 2a) were thoroughly rinsed in PBS and used as gate electrodes in the above described OECT platform.

Transfer characteristics, i.e., channel current (I_DS_) as a function of the gate voltage (V_GS_), were recorded accordingly using 2-channel source meter precision unit (B2902A, Keysight Technologies, Santa Rosa, CA, USA) by fixing the channel voltage (V_DS_) at −0.1 V and varying the V_GS_ between −0.1 V and 0.8 V (scan rate ΔV_GS_ = 0.02 V; scan time t = 3 s). One batch of PBS was used as a gate electrolyte. After each measurement, the device channel was rinsed three times in MilliQ water, with the aim of removing the residual saline content from the channel and, consequently, restoring the initial I_DS_ current value. The sensing performance was evaluated using the modulation parameter ΔI_DS_, defined as [(I_DS_(@V_GS_ = 0.8) − (I_DS_(@V_GS_ = −0.1)]/(I_DS_(@V_GS_ = −0.1). From the ΔI_DS_ vs. Hcy concentration curve, the Limit of Detection (LoD) was assessed as: LoD = 3σ/S, where σ is the standard deviation of the response (i.e., the standard deviation of the y-intercept of the regression line) and S is the slope of the regression line.

### 4.6. Pt Electrode Electrical Measurements

A Pt wire (purchased from Carlo Erba), 5 mm in length, was cleaned in hot acetone and IPA and used as gate electrode in the 3D-printed OECT platform.

Hcy solutions in the presence of BSA, with a concentration of Hcy ranging from 100 nM to 10 mM, were prepared following the dilution of Hcy in PBS and the subsequent addition of BSA in such a way as to achieve a fixed albumin concentration of 600 µM (physiological concentration). Each of the prepared solutions were used as gate electrolyte in Pt-gated OECT. The electrical characterization was again performed by acquiring transfer curves and extracting the ΔI_DS_ parameter, considering the same biasing conditions and rinsing procedure adopted for the Au-gated OECT.

In a complementary set of experiments, the OECT response in the presence of a bare Pt electrode was assessed using, as a gate electrolyte, some aliquots of the freshly-prepared Hcy:HCl:PBS solutions (with Hcy concentrations ranging between 100 nM and 1 mM) employed for the incubation of the gold electrode. For these measurements, the same previously adopted biasing conditions/rinsing procedures were utilized. 

## Figures and Tables

**Table 1:** Fit parameters extracted from EIS equivalent circuit model analysis.

Fit Parameters	SPE Au Bare	SPE Hcy-Functionalized
R_el_ (Ω)	30.6	32.4
R_ct_ (MΩ)	1.2	4.16
CPE (Sxs^n^)	5.9	-
Ideality factor, n	0.944	0.978
CPE_coverage_ (Sxs^n^)	-	1.68
R_el_^pores^ (KΩ)	-	511
C_DL_ (nF)	-	330

**Table 2:** List of the performed electrical tests.

Investigated Device	Employed Solutions for the Electrical Tests	Main Goal
Screen-printed electrode after incubation in Hcy/HCl/PBS	PBS	To confirm that gold incubation in Hcy/HCl/PBS solutions promotes thiolation of the gold electrode
OECT with Au gate incubated in Hcy/HCl/PBS at differnet concentrations	PBS	To explore the sensitivity of OECT vs. free Hcy
OECT with bare Pt gate	Hcy:PBS:BSA with different concentrations	To explore the sensitivity of OECT vs. protein-bound Hcy
OECT with bare Pt gate	Hcy:HCl:PBS with different concentrations	To investigate the occurrence of oxidative processes involving free Hcy at the platinum surface

## Data Availability

Not applicable.

## Supplementary Materials

The following are available online at www.mdpi.com/article/10.3390/ijms222111507/s1.

## Abbreviations

AJP	Aerosol Jet printing
BSA	Bovine Serum Albumin
CPE	Constant Phase Element
CV	Cyclic Voltammetry
EG	Ethylene Glycol
EGOFET	Electrolyte-Gated Organic Field-Effect Transistor
EIS	Electrochemical Impedance Spectroscopy
GOPS	3-glycidoxypropyltrimethoxysilano
Hcy	Homocysteine
HHcy	Hyperhomocysteinemia
HCl	Hydrochloric Acid
HPLC	High-Performance Liquid Chromatography
IPA	Isopropyl Alcohol
LoD	Limit of Detection
OECTs	Organic Electrochemical Transistors
PEDOT:PSS	poly(3,4-ethylenedioxythiophene)-polystyrene sulfonate
PBS	Phosphate-buffered Saline
POC	Point-of-Care
PDMS	Polydimethyl Siloxane
rHcy	reduced-free Homocysteine
SPEs	Screen-Printed Electrodes
tHcy	Total Homocysteine
