# Supplementary material for: Homocysteine Solution-Induced Response in Aerosol Jet Printed OECTs by Means of Gold and Platinum Gate Electrodes
Source: Int J Mol Sci. 2021 Oct 25;22(21):11507. doi: 10.3390/ijms222111507 (PMC8584102; doi:10.3390/ijms222111507)
Supplement: Supplementary file 1 [file ijms-22-11507-s001.zip › ijms-1427146-supplementary.pdf]

Article Supplementary Materials

Homocysteine solution-induced response in Aerosol Jet Printed OECTs by means of gold and platinum gate electrodes

Pasquale D'Angelo[1,#], Mario Barra[2,#], Patrizia Lombari[3,4,#], Annapaola Coppola[3], Davide Vurro[1], Giuseppe Tarabella[5], Simone Luigi Marasso[1], Margherita Borriello[3], Federico Chianese[6], Alessandra F. Perna[4], Antonio Cassinese[7,6,2*] and Diego Ingrosso[3]

[1] IMEM-CNR, Parco Area delle Scienze 37/A, I-43124 Parma e-mail pasquale.dangelo@imem.cnr.it
[2] CNR-SPIN, c/o Dipartimento di Fisica "Ettore Pancini", P.le Tecchio, 80, I-80125 Naples, Italy e-mail mario.barra@spin.cnr.it
[3] Department of Precision Medicine, University of Campania "Luigi Vanvitelli", via L. De Crecchio, 7 - 80138 Naples, Italy e-mail patrizia.lombari@unicampania.it ; annapaola.coppola@unicampania.it; diego.ingrosso@unicampania.it
[4] Department of Translational Medical Science University of Campania "Luigi Vanvitelli", via Via Pansini, Bldg. 17 - 80131 Naples, Italy e-mail patrizia.lombari@unicampania.it ; alessandra.perna@unicampania.it
[5] Camlin Italy Srl, Via Budellungo 2, I-43124, Parma e-mail
[6] Physics Department, University of Naples 'Federico II', P.le Tecchio, 80, I-80125 Naples, Italy e-mail antonio.cassinese@unina.it.
[7] Istututo Nazionale di Fisica Nucleare, Sezione di Napoli, P.le Tecchio, 80, I-80125 Naples, Italy e-mail antonio.cassinese@unina.it.

* Correspondence: A.C.[6,2] antonio.cassinese@unina.it; Tel.: ; A.F.P. alessandra.perna@unicampania.it; Tel.: +390815666822
# These Authors equally contributed to this work.

Citation: D'Angelo, P.; Barra, M.; Lombari, P. Homocysteine solution-induced OECT response by means of gold and platinum gate electrodes. *Int. J. Mol. Sci.* **2021**, *22*, 11507. https://doi.org/10.3390/ijms222111507

Academic Editor: Firstname Lastname

Received: date
Accepted: date
Published: date

Publisher's Note: MDPI stays neutral with regard to jurisdictional claims in published maps and institutional affiliations.

Copyright: © 2021 by the authors. Submitted for possible open access publication under the terms and conditions of the Creative Commons Attribution (CC BY) license (https://creativecommons.org/licenses/by/4.0/).

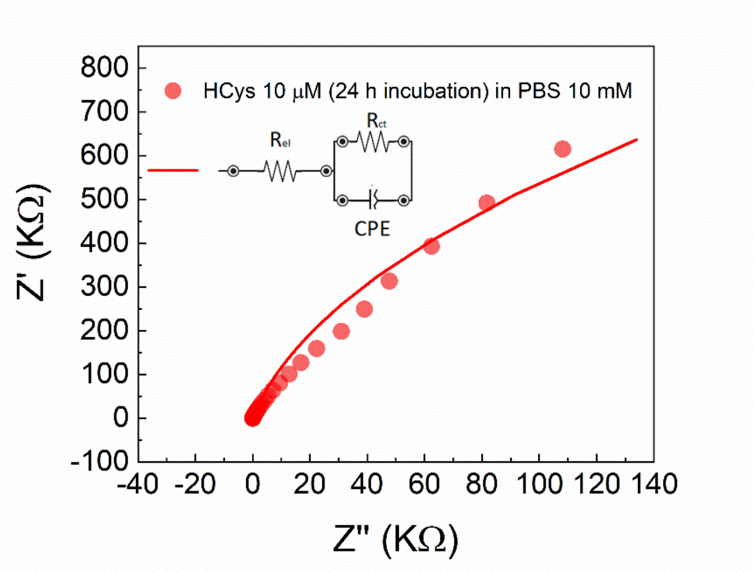

Figure S1. Nyquist plot (red symbols) for the gold ~~WE~~ working electrode after the incubation in HCy:HCl:PBS (HCy 10 μM) solutions; the red line is the fitting curve related to a Randles equivalent circuit.

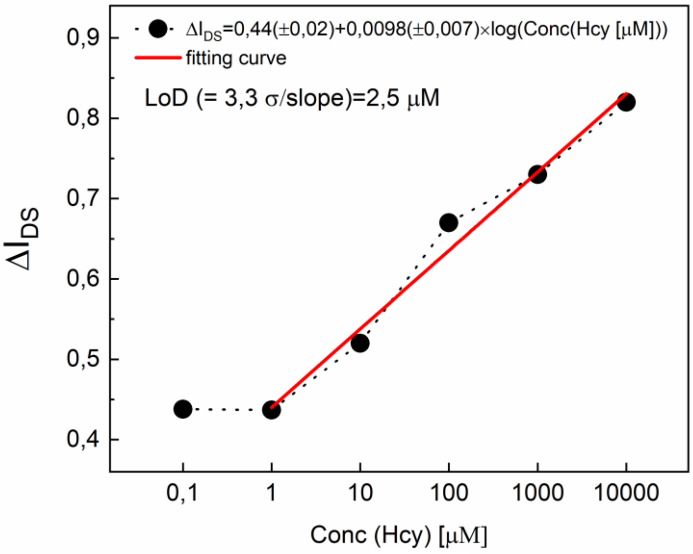

(a)

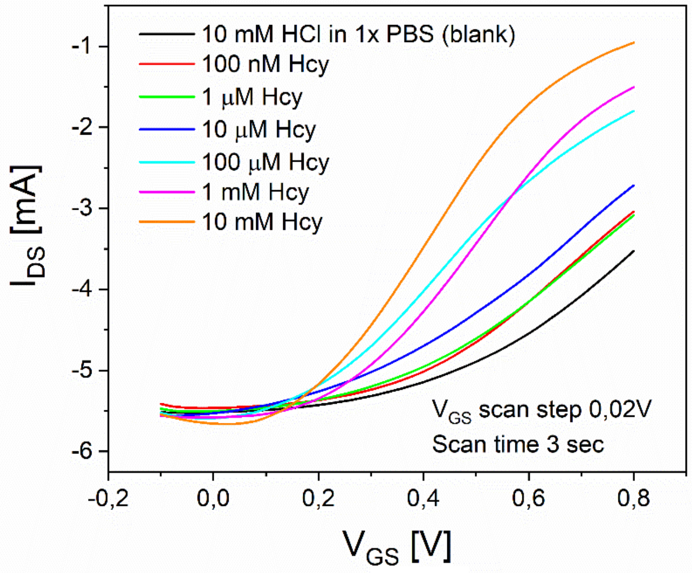

(b)

Figure S2. (a) OECT transfer curves recorded using a platinum electrode and PBS:HCl:PBS solutions (used for gold electrodes decorations by Hcy biothiols) at different Hcy (and HCl) concentrations, as electrolyte. The measurement achieved using the stock solution (with 10 mM of Hcy) as electrolyte has been also included. (b) modulation parameter ΔI_{DS} extracted from all the recorded transfer curves in panel (a), as a function of the Hcy concentration in Hcy:HCl:PBS solutions.

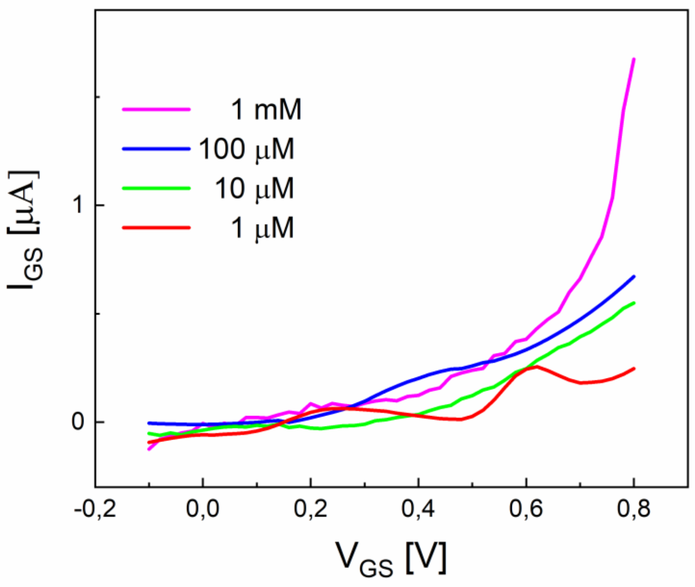

Figure S3. Gate currents as a function of the gate voltage for representative transfer curves reported in Figure 4a in 2.2 subsection

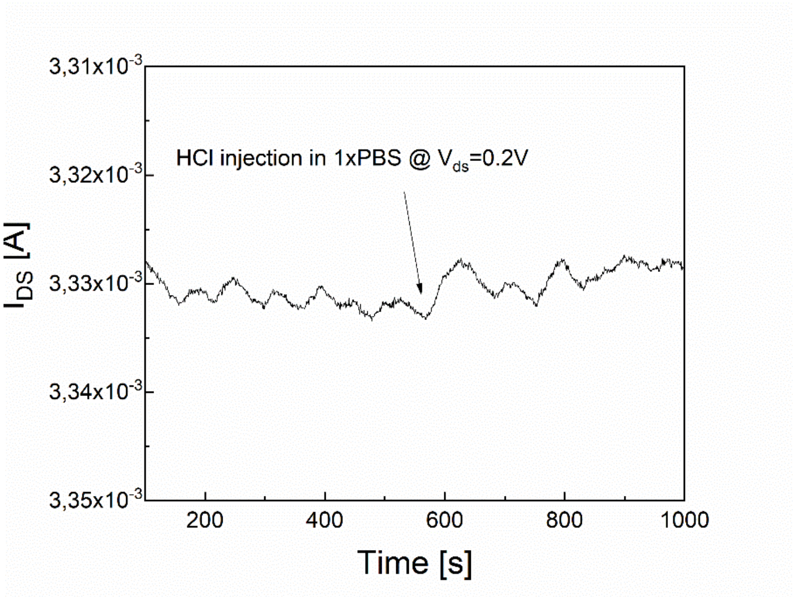

Figure S4. Effect of HCl (10 N) injection in 1x PBS gate electrolyte at t=550 s (black arrow is a guide to the eye)
